# Identification of the ferroptosis-related long non-coding RNAs signature to improve the prognosis prediction and immunotherapy response in patients with NSCLC

**DOI:** 10.1186/s12920-021-01133-4

**Published:** 2021-12-03

**Authors:** Meng Li, Yanpeng Zhang, Meng Fan, Hui Ren, Mingwei Chen, Puyu Shi

**Affiliations:** 1 Department of Respiratory and Critical Care Medicine, The First Affiliated Hospital of Xi’an Jiao Tong University, No. 277, Yanta West Road, Xi’an, 710061 Shaanxi China; 2 Department of Talent Highland, The First Affiliated Hospital of Xi’an Jiao Tong University, No. 277, Yanta West Road, Xi’an, 710061 Shaanxi China; 3 Department of Thoracic Surgery, The First Affiliated Hospital of Xi’an Jiao Tong University, No. 277, Yanta West Road, Xi’an, 710061 Shaanxi China; 4 Department of Center for Translational Medicine, The First Affiliated Hospital of Xi’an Jiao Tong University, No. 277, Yanta West Road, Xi’an, 710061 Shaanxi China

**Keywords:** Non-small cell lung cancer, Ferroptosis, Long non-coding RNAs, Prognostic, Signature

## Abstract

**Background:**

Non-small cell lung cancer (NSCLC) is the most prevalent type of lung carcinoma with an unfavorable prognosis. Ferroptosis is involved in the development of multiple cancers. Whereas, the prognostic value of ferroptosis-related lncRNAs in NSCLC remains uncertain.

**Methods:**

Gene expression profiles and clinical information of NSCLC were retrieved from the TCGA database. Ferroptosis-related genes (FRGs) were explored in the FerrDb database and previous studies, ferroptosis-related lncRNAs (FRGs-lncRNAs) were identified by the correlation analysis and the LncTarD database. The differentially expressed FRGs-lncRNAs were screened and FRGs-lncRNAs associated with the prognosis were explored by univariate Cox regression analysis and Kaplan–Meier survival analysis. Then, an FRGs-lncRNAs signature was constructed and verified by the Lasso-penalized Cox analysis. Finally, the potential correlation between risk score, immune checkpoint genes, and chemotherapeutic sensitivity was further investigated.

**Results:**

129 lncRNAs with a potential regulatory relationship with 59 differentially expressed FRGs were found in NSCLC, of which 10 were related to the prognosis of NSCLC (*P* < 0.05). 9 prognostic-related FRGs-lncRNAs were used to construct the prognostic model and stratify NSCLC patients into high- and low-risk groups. A worse outcome was found in patients with high risk (*P* < 0.05). Moreover, a good predictive capacity of this signature in predicting NSCLC prognosis was confirmed. Additionally, 45 immune checkpoint genes and 4 chemotherapeutics drugs for NSCLC were identified to be correlated with the risk score.

**Conclusion:**

A novel FRGs-lncRNAs signature was successfully constructed, which may contribute to improving the management strategies of NSCLC.

**Supplementary Information:**

The online version contains supplementary material available at 10.1186/s12920-021-01133-4.

## Introduction

Lung cancer ranks top in the incidence of malignancies and imposes an enormous socio-economic burden worldwide [1]. Non-small-cell lung cancer (NSCLC) is the most prevalent subtype of primary lung cancer, among which adenocarcinoma (LUAD) as well as squamous cell carcinoma (LUSC) is the leading histological type [2, 3]. Although there have been breakthroughs in the targeted therapy and immunotherapy of lung cancer, the long-time survival rate of NSCLC remains unsatisfactory [4–6], and most patients will inevitably develop drug resistance. Since most anti-tumor drugs play a therapeutic role by inducing apoptosis of cancer cells, it is of great significance to pinpoint novel cell death pathways in NSCLC and discover new directions for identifying treatment strategies and evaluating prognosis.

Ferroptosis, a newly discovered mode of iron-dependent regulated cell death unlike apoptosis, pyroptosis, autophagy and necrosis, is mainly caused by the iron accumulation-mediated lipid peroxidation and exhibits peculiar morphology, genetics, as well as biochemistry features [7]. The development of disease and organisms and anti-tumor drug resistance has also been verified to be affected by ferroptosis [8, 9]. Long non-coding RNAs (lncRNAs) are a kind of RNA with more than 200 bases in length. Although lncRNAs lack protein-coding ability, it is still considered a target for gene therapy of cancer since it is involved in the tumor occurrence, development as well as metastasis by modulating gene expression at chromatin, transcriptional, and post-transcriptional levels [10, 11]. Additionally, some lncRNAs were found to inhibit ferroptosis by acting as competitive endogenous RNA to prevent oxidation in various cancers including lung cancer [12, 13]. Metallothionein 1D pseudogene, a lncRNA, was found to sensitize erastin-induced ferroptosis in NSCLC by modulating the miR-365a-3p/NRF2 axis [14]. A G3BP1-interacting lncRNA was demonstrated to promote ferroptosis via nuclear sequestration of p53 in lung carcinoma [15]. Recently, several studies have constructed some prognostic signatures in many cancers, including LUAD [16], head and neck squamous cell carcinoma [17], bladder [18], and colon cancer [19], by exploring the prognostic ferroptosis-related lncRNAs to assist clinicians to evaluate the prognosis of patients. Whereas, the signature of ferroptosis-related lncRNAs and its association with prognosis in NSCLC has not been systematically evaluated.

Herein, we explored the expression of ferroptosis-related genes (FRGs) and ferroptosis-related lncRNAs (FRGs-lncRNAs) in NSCLC and further investigate the relationship between these lncRNAs and the prognosis in NSCLC based on the Cancer Genome Atlas (TCGA) database. Subsequently, a prognostic model was constructed on the basis of FRGs-lncRNAs in the training set and verified by internal and external validation. Furthermore, the relationship between risk score, clinicopathological features, immune checkpoint genes (ICGs), and chemotherapeutics sensitivity was further elucidated in NSCLC.

## Materials and methods

### Data acquisition and processing

Gene expression profiles and clinical features of TCGA-LUAD (510 tumors and 58 normal) and TCGA-LUSC (496 tumors and 49 normal) were obtained from UCSC Xena (https://xena.ucsc.edu/). The expression profiles of LUAD and LUSC were combined as NSCLC expression matrix, and a total of 1093 patients (986 tumors and 107 normal) were eventually included in the present study after the batch correction by using ComBat function of the R “sva” package. No ethics committee approval and informed consent of patients were required in this study since the data were obtained from a public database.

### Identification of FRGs

259 FRGs (Additional file 2: Table S1) were explored from the “Marker”, “Driver” and “Suppressor” modules of the FerrDb database (http://www.zhounan.org/ferrdb/) [20] and previous studies [8, 21–23], among which 241 were found to be expressed in NSCLC on the basis of available mRNA expression data. The expression profiles of these FRGs were extracted to identify the differentially expressed FRGs (DE-FRGs) between normal and tumor by the “limma” package of R. |log2FC|> 1and FDR < 0.05 is considered to be significant.

### Functional enrichment analysis

“ClusterProfiler” of R was utilized to conduct Gene Ontology (GO) as well as Kyoto Encyclopedia of Genes and Genomes (KEGG) analysis to investigate the biological functions and signaling pathways affected by these DE-FRGs [24–26].

### Screening of FRGs-lncRNAs

Pearson correlation analysis was conducted to identify the potential lncRNAs correlated with DE-FRGs based on the TCGA database. ∣R|> 0.5 and *P* < 0.001 were considered a strong correlation. In addition, the lncRNAs that have a regulatory relationship with DE-FRGs and expressed in NSCLC were further explored and screened in the LncTarD database (http://bio-bigdata.hrbmu.edu.cn/LncTarD/) [27]. Then, lncRNAs that have an expression and regulatory relationship with DE-FRGs in TCGA and LncTarD database were unionized to obtain candidate FRGs-lncRNAs. The “survival” package, univariate Cox regression analysis, and Kaplan–Meier (K-M) survival method were conducted to investigate FRGs-lncRNAs associated with the prognosis in NSCLC.

### FRGs-lncRNAs prognostic model construction

986 NSCLC patients were randomly separated into training and verification set at a ratio of 7:3 by R. Lasso-penalized Cox regression analysis was performed to establish an FRGs-lncRNAs prognostic model in the training set using the R “glmnet” package [28, 29]. The risk score of each sample was calculated on the basis of the normalized expression levels and the corresponding Lasso’s coefficient of the FRGs-lncRNAs [Risk score = ∑exp(i)*coef(i)], and patients were further categorized as high- and low-risk groups in accordance with the median risk score. Then “ggplot2” package of R was performed to draw the survival scatter plot. K-M survival curve and receiver operational characteristic (ROC) curves were generated to evaluate the relationship between risk score and prognosis, and the predictive capacity of the signature. Additionally, the expression levels of FRGs-lncRNAs of each sample in the two risk groups were uncovered by the “heatmap” package. Finally, the results were further identified in the internal and external validation groups.

### Predictive nomogram construction

Wilcoxon test was performed to investigate the potential relationship between risk score and multiple clinical features (EGFR mutation, ALK-EML4 rearrangement, age, gender, stage, and TNM stage). *P* < *0*.05 was considered to be significant. Subsequently, the independent prognostic factors were investigated by univariate and multivariate Cox regression analysis and visualized the results by R “forestplot” package. Finally, a nomogram was established integrating the independent risk factors (T, N and Risk score) and M stage with important clinical prognostic significance for predicting 1, 3, and 5‐year overall survival (OS) of NSCLC patients by using the “rms” package of R, and the prediction accuracy of which was further evaluated by the calibration and ROC curve.

### Correlation analysis between risk score, ICGs and chemotherapeutic drugs sensitivity

79 ICGs were explored from the previous literature [30], of which 66 were expressed in NSCLC. Student t-test was used for difference analysis, and the top 10 ICGs with significant differences were shown as boxplots. The correlation between risk score and sensitivity of chemotherapeutics commonly used for NSCLC treatment, including cisplatin, etoposide, docetaxel, gefitinib, erlotinib, gemcitabine, paclitaxel, was evaluated by using Wilcoxon signed-rank test of the R package “pRRophetic” and “ggplot2”. *P* < 0.05 is considered a significant correlation.

### Statistical analysis

Perl and R (4.0.1) were utilized for data processing and statistical analysis. Benjamini–Hochberg method was performed to identify the DE-FRGs. Pearson correlation analysis were conducted to explore the lncRNAs associated with DE-FRGs. FRGs-lncRNAs associated with prognosis were explored by univariate Cox regression tests and K-M survival analysis in NSCLC. An FRGs-lncRNAs prognostic model was generated by the Lasso-penalized Cox regression analysis and further evaluated the predictive capacity by K-M survival and ROC curve analysis. Independent predictors of OS in NSCLC were identified by multivariate Cox regression analysis. *P* < 0.05 was considered significant.

## Results

### Identification and enrichment analysis of FRGs in NSCLC

**Fig. 1:**
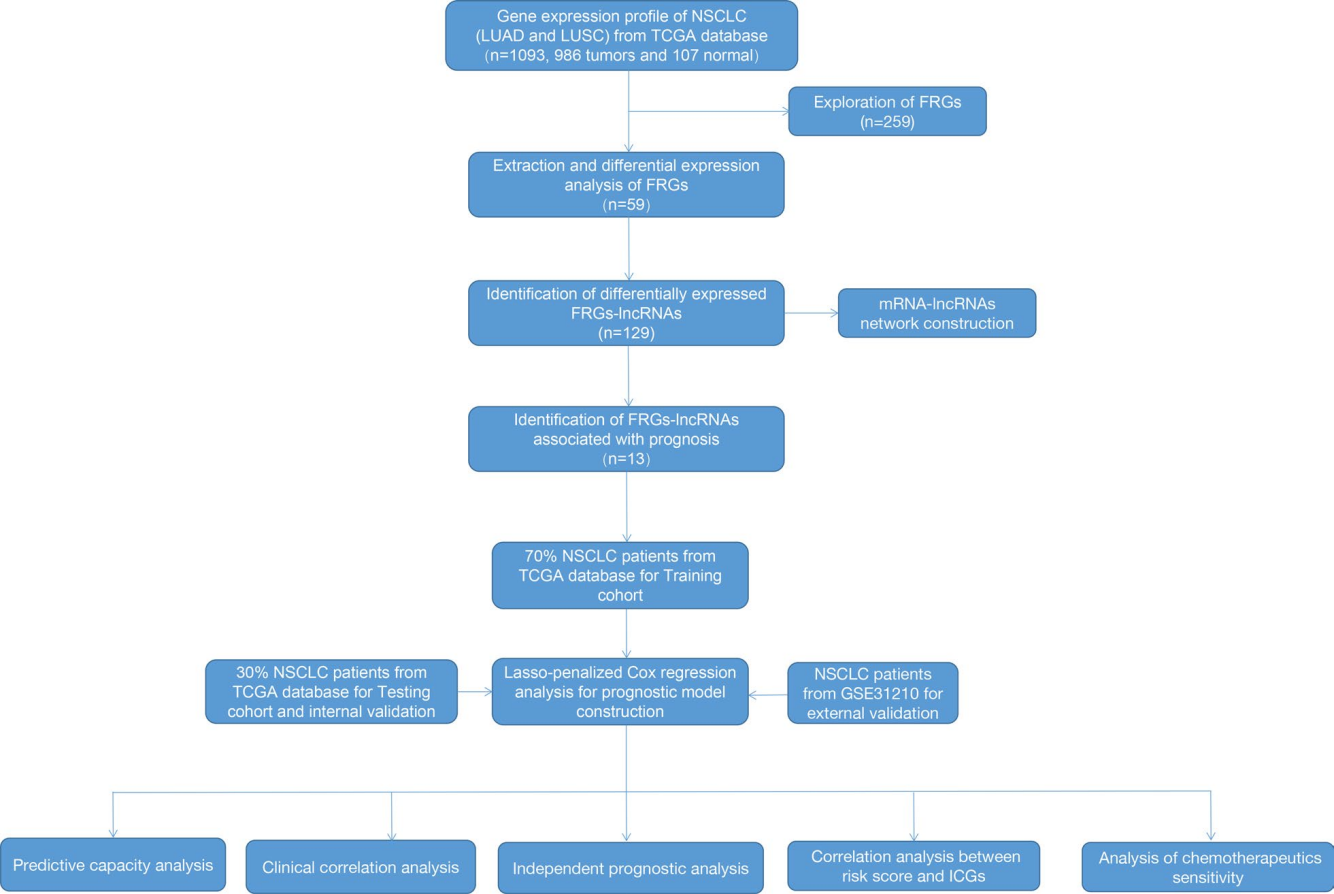
Flowchart of the present study. NSCLC, non-small cell lung cancer; LUAD, lung adenocarcinoma; LUSC: lung squamous cell carcinoma; FRGs, ferroptosis-related genes; ICGs, immune checkpoint genes

**Fig. 2:**
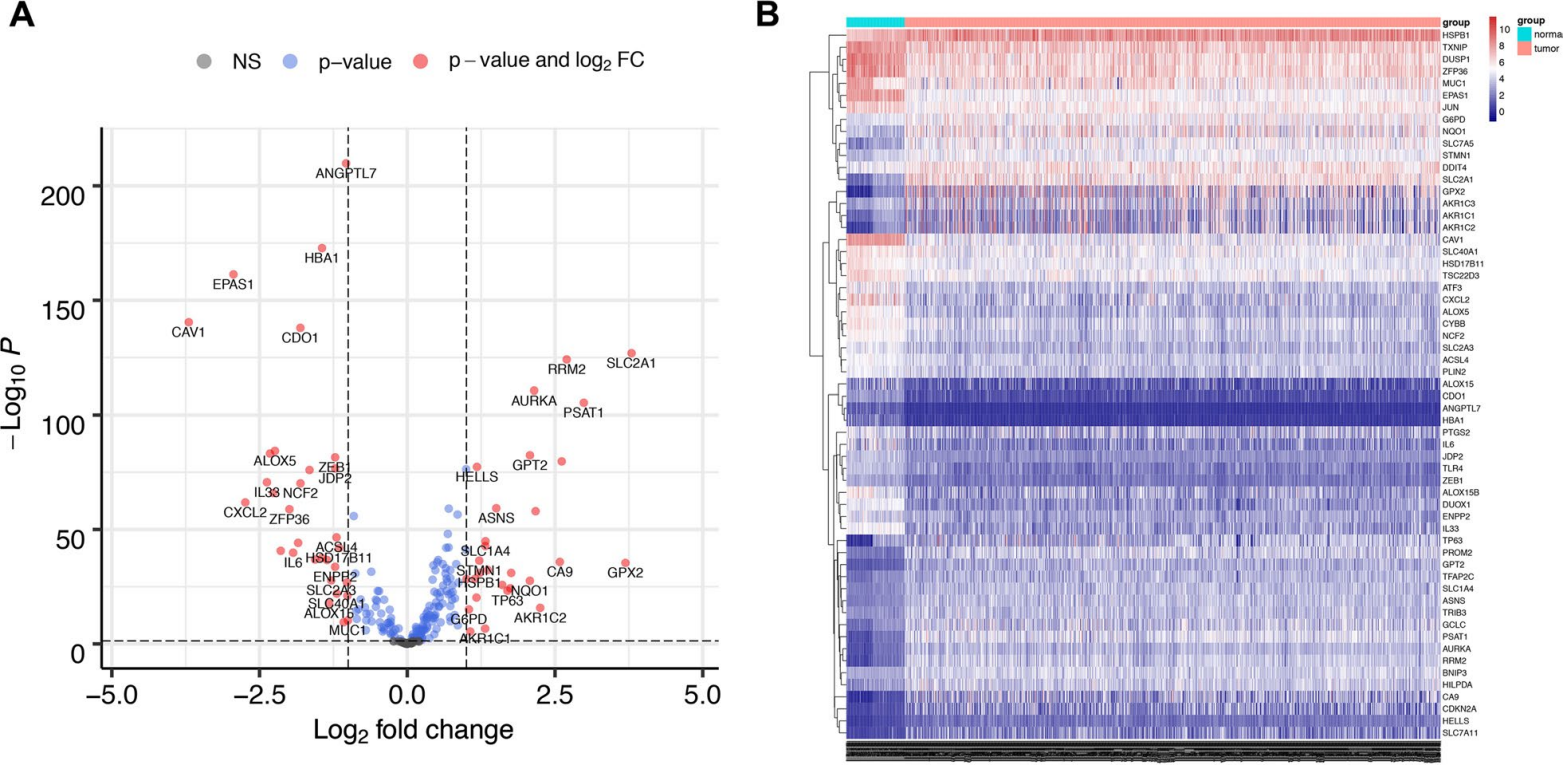
Identification of the FRGs in patients with NSCLC. The DE-FRGs in NSCLC were shown in the volcano plot (**A**) and heatmap (**B**). FDR < 0.05 and |log2FC|≥ 1 were considered significant. The gray dots in **A** represent the FRGs with no statistical difference, the blue dots represent the FRGs with FDR < 0.05, and the red dots represent genes with FDR < 0.05 and |log2FC|≥ 1. FRGs, ferroptosis-related genes; DE-FRGs, differentially expressed FRGs; NSCLC, non-small cell lung cancer; FDR, false discovery rate

**Fig. 3:**
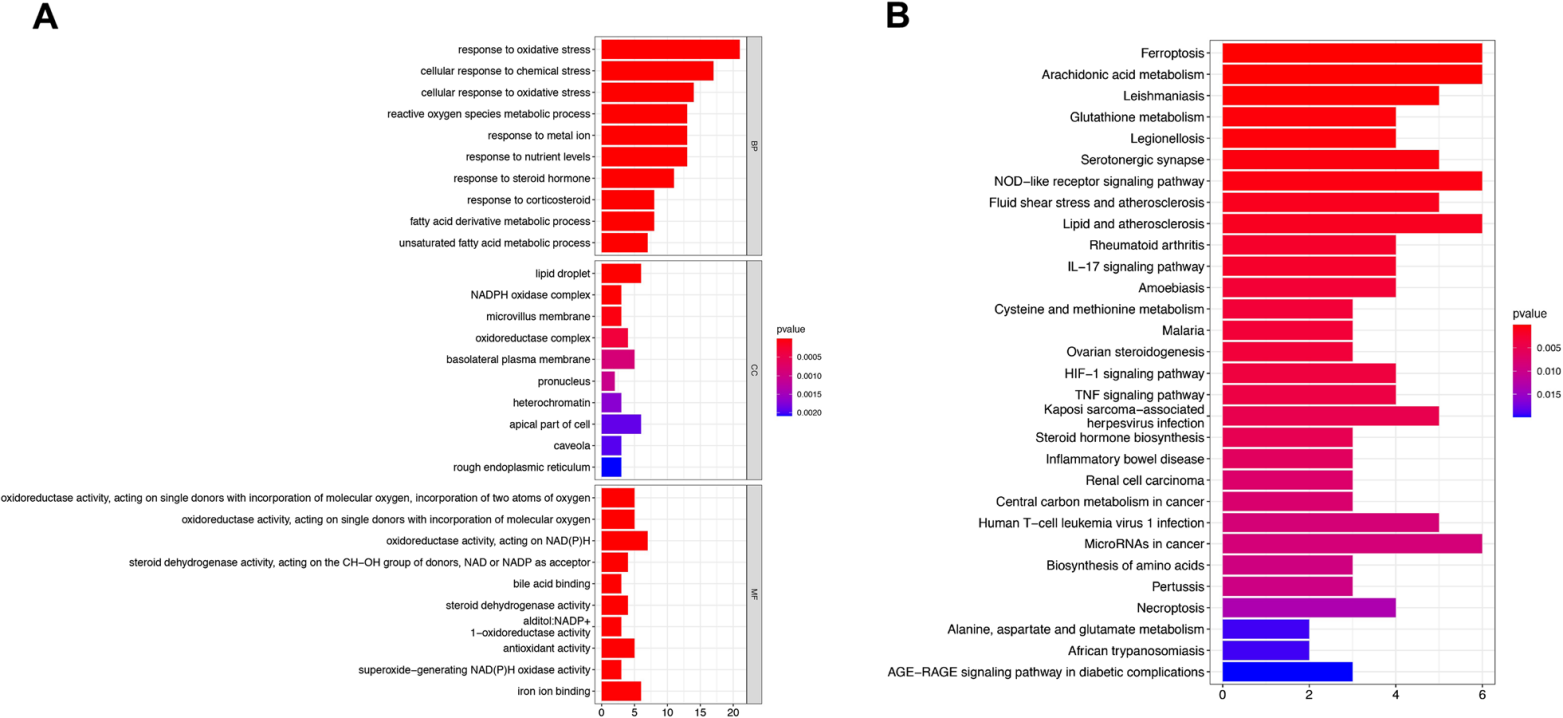
GO (**A**) and KEGG [24–26]. (**B**) analysis of the DE-FRGs. The X-axis represents the number of DE-FRGs enriched on each GO and KEGG. The color of the cuboid represents the significance of enrichment. GO, Gene Ontology; KEGG, Kyoto Encyclopedia of Genes and Genome; FRGs, ferroptosis-related genes; DE-FRGs, differentially expressed FRGs; BP, biological process; CC, cellular component; MF, molecular function

986 patients with NSCLC and 107 healthy control from the TCGA database were enrolled in the present study. 259 FRGs were explored from the FerrDb database and previous studies, among which 241 were found to be expressed in NSCLC (Additional file 2: Table S1). The process of our research is shown in Fig. 1. Herein, we found 59 FRGs were significantly differentially expressed between normal and NSCLC patients (28 downregulated and 31 upregulated, Fig. 2A, B). The DE-FRGs were determined mainly involved in biological pathways related to the response to oxidative stress, lipid droplet, and oxidoreductase activity biological function by GO analysis (Fig. 3A). KEGG enrichment analysis revealed that these DE-FRGs also participate in ferroptosis, arachidonic acid metabolism, glutathione metabolism, and NOD-like receptor signaling pathway (Fig. 3B).

### Identification of the ferroptosis-related lncRNAs in NSCLC

**Fig. 4:**
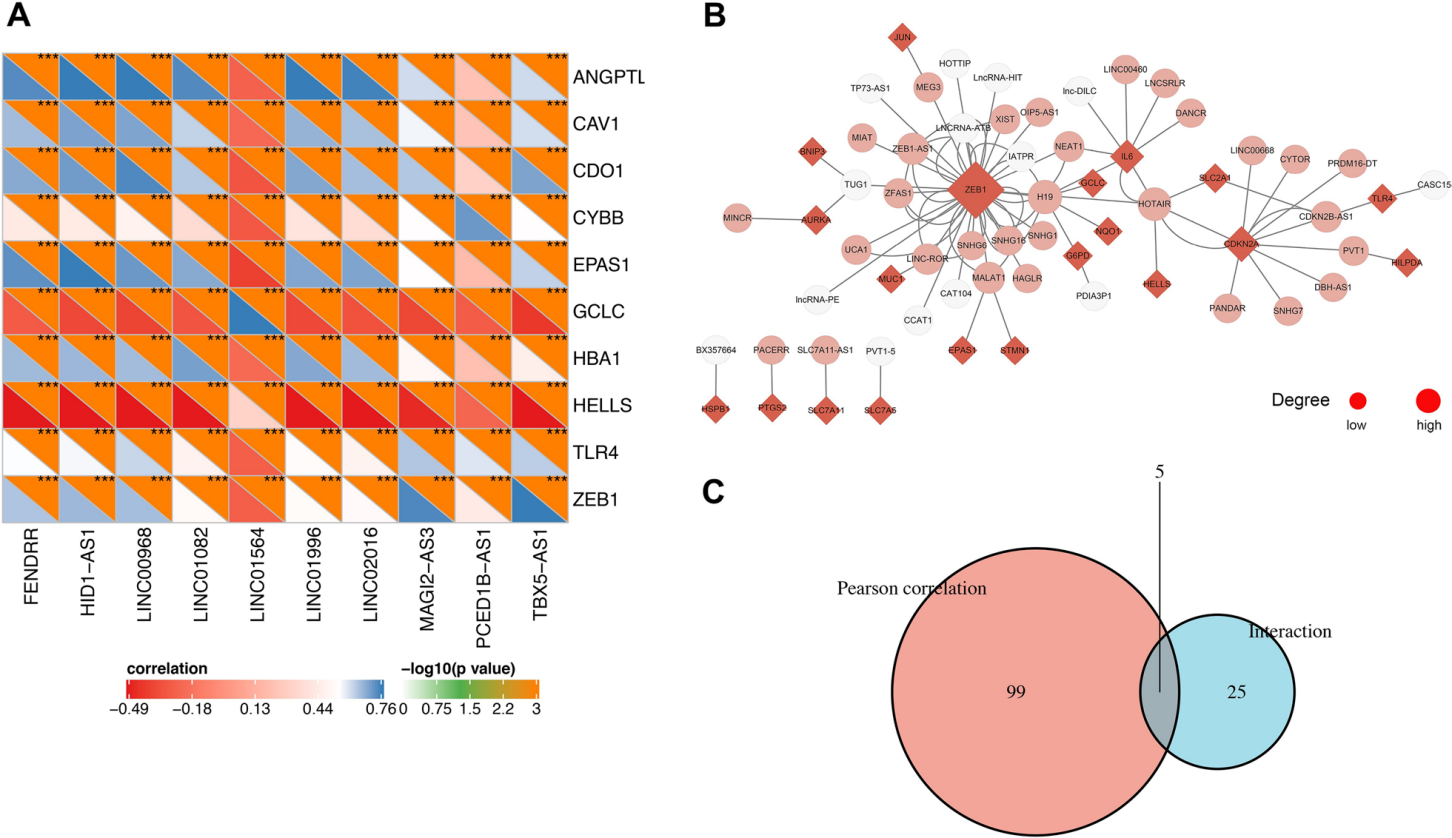
Identification of the FRGs-lncRNAs in NSCLC. (**A**) Correlation heatmap of the top 10 FRGs and lncRNAs based on TCGA database. The criteria of correlation analysis: |R|> 0.5 and *P* < 0.001. (**B**) Interaction network between DE-FRGs and lncRNAs in LncTarD database. The DE-FRGs and lncRNAs are shown as the red diamond and pink circle, respectively. The white circles represent lncRNAs that are not expressed in NSCLC. The size of the dot is positively correlated with the degree of correlation. (**C**) The union set of the DE-FRGs related lncRNAs in the TCGA database and LncTarD database were shown as a Venn diagram. FRGs, ferroptosis-related genes; lncRNAs: long non-coding RNAs; FRGs-lncRNAs, FRGs-related lncRNAs; TCGA: the Cancer Genome Atlas; NSCLC, non-small cell lung cancer; DE-FRGs: differentially expressed FRGs

**Fig. 5:**
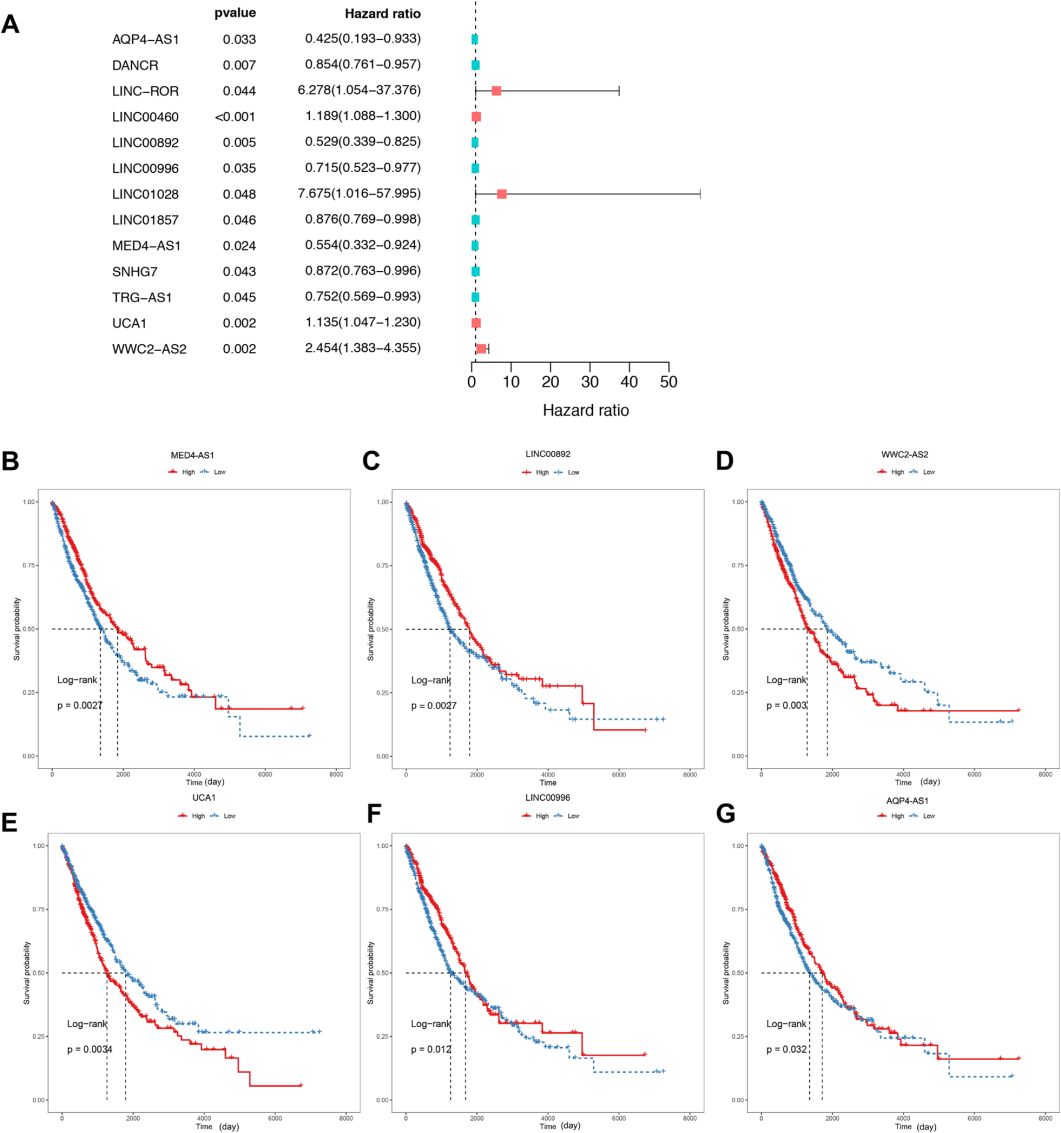
Identification of the FRGs-lncRNAs associated with prognosis. Univariate Cox regression analysis (**A**) and Kaplan–Meier survival analysis (**B–G**) identified the FRGs-lncRNAs associated with the prognosis of NSCLC. The red and blue curve in **B–G** represents the patients with high and low expression of FRGs-lncRNAs, which was classified by the median expression level of the FRGs-lncRNAs. NSCLC, non-small cell lung cancer; FRGs, ferroptosis-related genes; FRGs-lncRNAs: FRGs related lncRNAs

104 FRGs-lncRNAs were identified by Pearson correlation analysis (|R|> 0.5, *P* < 0.001) and the top 10 of which were shown in Fig. 4A. Next, 44 lncRNAs that have a regulatory relationship with DE-FRGs were explored in the LncTarD database, and 30 of them were determined to be expressed in NSCLC. The interaction network between DE-FRGs and lncRNAs in the LncTarD database were shown in Fig. 4B. Finally, 129 candidate FRGs-lncRNAs were collected in this study by unifying the two methods (Fig. 4C, Additional file 2: Table S2). Then, 13 FRGs-lncRNAs associated with NSCLC prognosis were identified by univariate Cox analysis (Fig. 5A, Additional file 2: Table S3), among which, 10 lncRNAs also showed significant results in K-M analysis and were selected for the prognostic model construction (Additional file 2: Table S4). The survival curves of the first 6 lncRNAs were demonstrated in Fig. 5B–G and the emperical cumulative density function (ecdf) plots of all 10 lncRNAs were shown in Additional file 1: Figure S1 (*P* < 0.05).

### Construction of the prognostic model based on ferroptosis-related lncRNAs

**Fig. 6:**
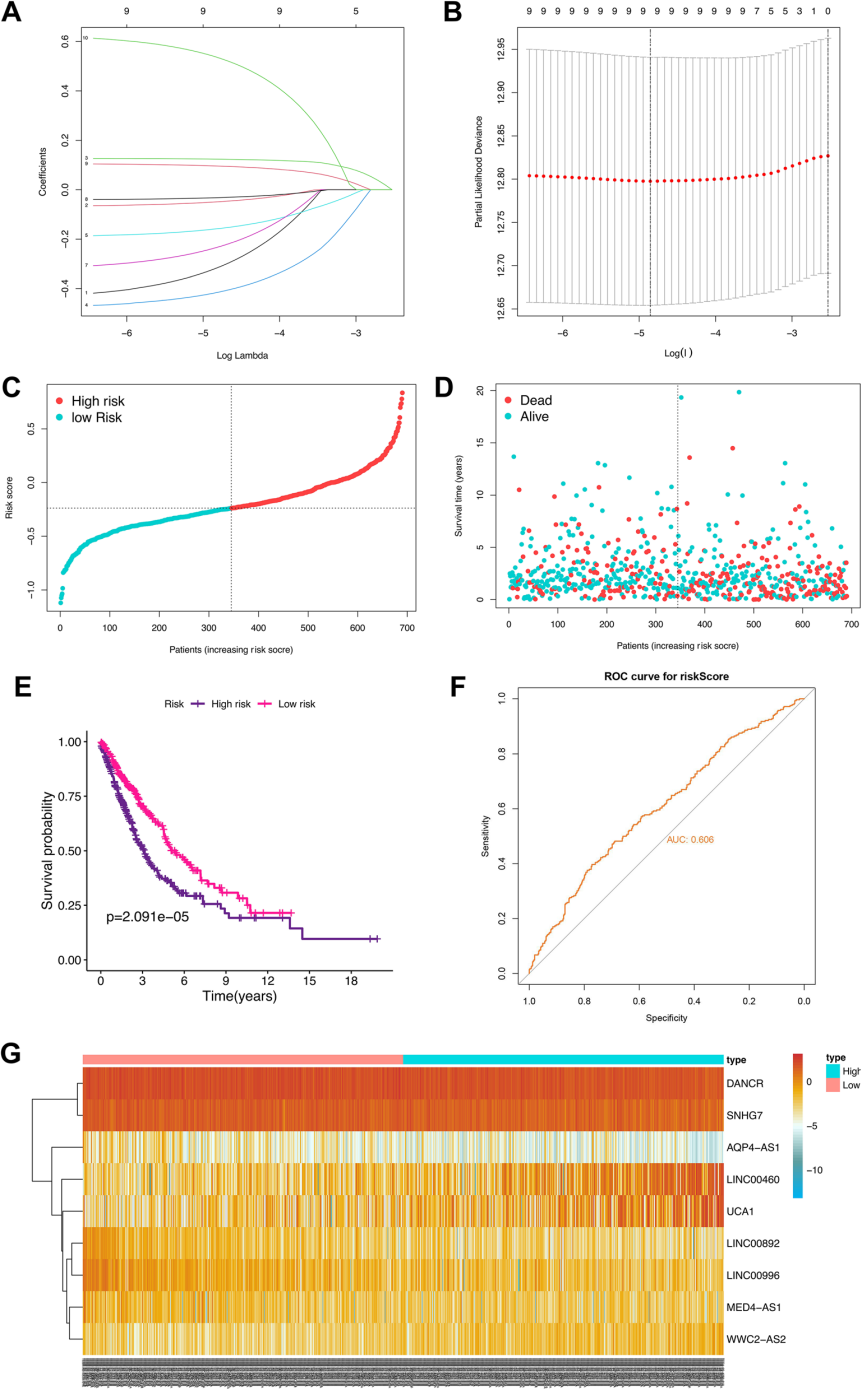
Construction of the FRGs-lncRNAs signature in the training group. (**A**) Lasso coefficients profiles of the 10 FRGs-lncRNAs. (**B**) Lasso regression analysis obtained 9 prognostic FRGs-lncRNAs. Distribution (**C**) and survival status plot (**D**) of NSCLC patients based on the median risk score. Kaplan–Meier survival (**E**) and ROC curve analysis (**F**) of the FRGs-lncRNAs signature in the training group. (**G**) Heatmap of the expression profiles of the FRGs-lncRNAs in low- and high-risk groups. Each curve in **A** represents the changing track of Lasso’s coefficients of the 10 prognosis-related FRGs-lncRNAs, respectively. The Y-axis represents the Lasso’s coefficient, the lower and upper X-axis represents log(λ), and the number of FRGs-lncRNAs with non-zero coefficients, respectively. In **B**, the Y-axis represents partial likelihood deviance, the lower and upper X-axis represents log(λ) and the number of FRGs-lncRNAs corresponding to the different log(λ), respectively. The upper X-axis corresponding to the smallest partial likelihood deviance is the best number of FRGs-lncRNAs included in the model. NSCLC, non-small cell lung cancer; lncRNAs: long non-coding RNAs; FRGs, ferroptosis-related genes; FRGs-lncRNAs: FRGs related lncRNAs; ROC, receiver operating characteristic

986 NSCLC patients were categorized into training and testing cohorts randomly at a ratio of 7:3, and 9 of the 10 prognostic FRGs-lncRNAs (AQP4-AS1, DANCR, LINC00460, LINC00892, LINC00996, MED4-AS1, SNHG7, UCA1, and WWC2-AS2) were eventually used for prognostic signature construction (Fig. 6A, B, Additional file 2: Table S5). Then, patients were classified into a high and low-risk group in accordance with the median of risk score [Risk score = exp(AQP4-AS1) * − 0.268 + exp(DANCR) * − 0.041 + exp(LINC00460) * 0.122 + exp(LINC00892) * − 0.390 + exp(LINC00996) * − 0.144 + exp(MED4-AS1) * − 0.198 + exp(SNHG7) * − 0.028 + exp(UCA1) * 0.092 + exp(WWC2-AS2) * 0.497] and the proportion of dead patients is higher in the patients with high-risk score (Fig. 6C, D). Additionally, the K-M plot and ROC curve analysis illustrated a worse prognosis in the NSCLC patients with the high-risk and a good predictive capacity of the signature, respectively (*P* < *0*.01, AUC = 0.606, Fig. 6E, F). The expression levels of the 9 FRGs-lncRNAs between the two risk groups are presented in Fig. 6G and Additional file 1: Figure S2.

### Validation of the prognostic signature

**Fig. 7:**
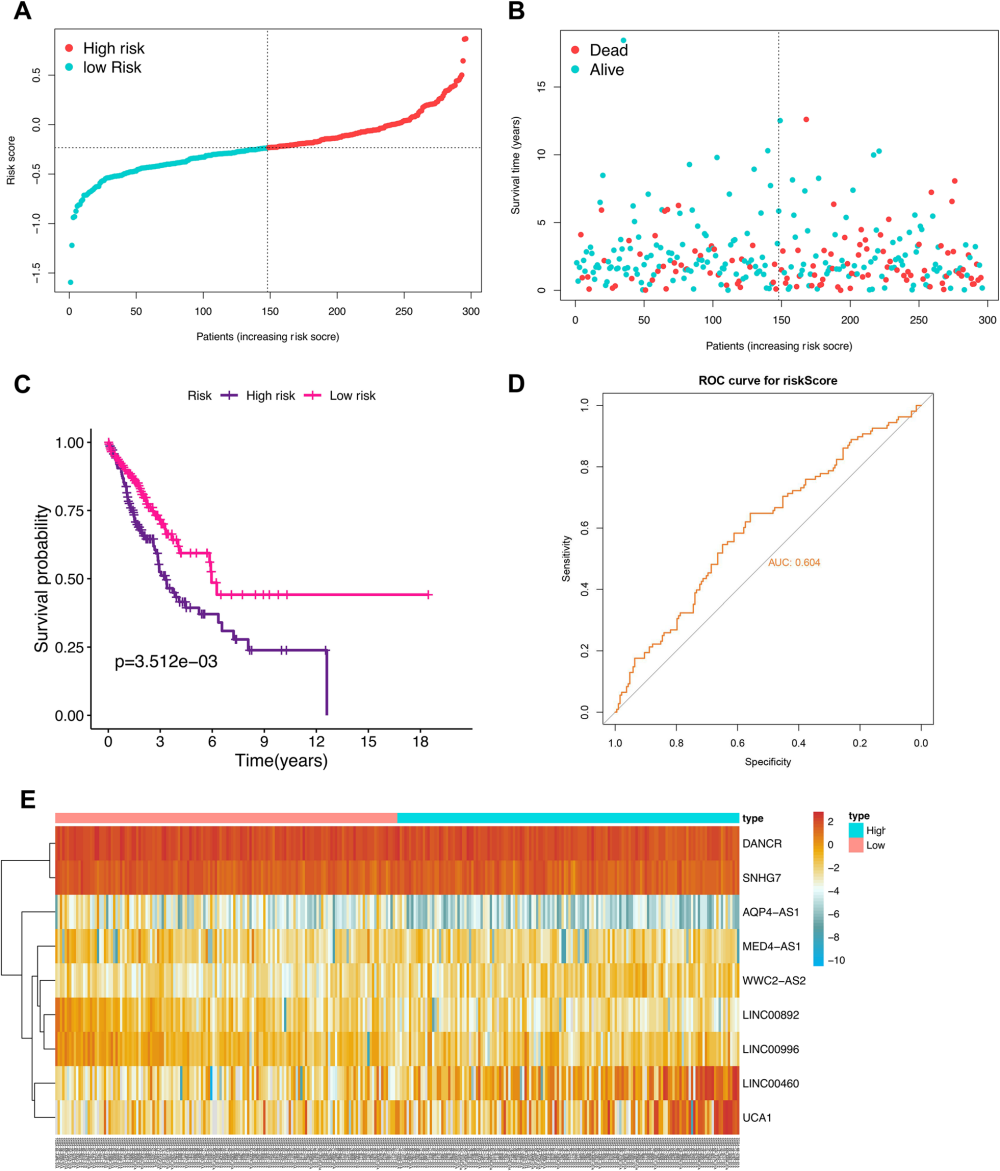
Validation of the FRGs-lncRNAs signature. Distribution (**A**) and survival status plot (**B**) of NSCLC patients based on the median risk score. Kaplan–Meier survival (**C**) and ROC curve analysis (**D**) of the FRGs-lncRNAs signature in the internal testing group. (**E**) Heatmap of the expression profiles of the FRGs-lncRNAs in low- and high-risk groups. NSCLC, non-small cell lung cancer; lncRNAs: long non-coding RNAs; FRGs, ferroptosis-related genes; FRGs-lncRNAs: FRGs related lncRNAs; ROC, receiver operating characteristic

To evaluate the robustness of the prognostic model, the patients from the testing cohort were also grouped according to the median of the same risk score (Fig. 7A), and the survival status scatter plot, K-M plot and ROC curve analysis demonstrated the similar results to the training group (*P* < *0*.01, AUC = 0.604, Fig. 7B–D). The expression trend of the 9 FRGs-LncRNAs between the two risk groups of the testing cohort was similar to that of the training group (Fig. 7E, Additional file 1: Figure S3).

In addition, external validation was also carried out in GES31210 to further verify the effectiveness of this prognostic model. Here, 8 lncRNAs (without MED4-AS1) were utilized for validation since MED4-AS1 expression was not found in NSCLC in this cohort. The results of the survival status scatter plot and heatmap in this cohort were similar to those of the training group (Additional file 1: Figure S4A-B, Figure S4E). and ROC curve and K-M plot analysis also illustrated that the good prediction performance of this prognostic signature and the correlation between the risk score based on these 8 lncRNAs and the prognosis of NSCLC (*P* = 0.016, AUC = 0.616 Additional file 1: Figure S4C and D).

### Correlation analysis between risk score and clinical characteristics and the construction of nomogram

**Fig. 8:**
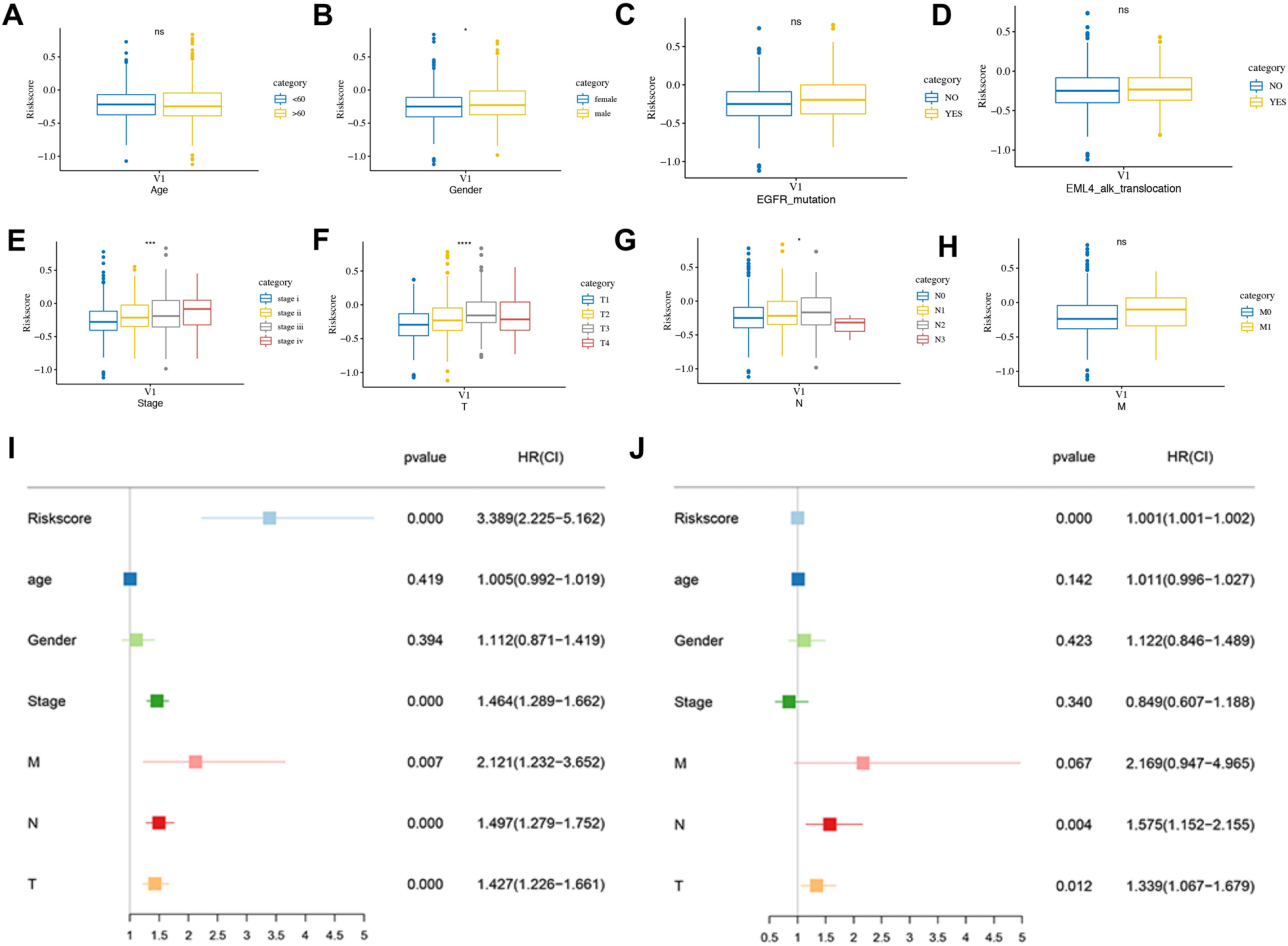
Correlation analysis and independent prognostic analysis. Correlation analysis between risk score and Age (**A**), Gender (**B**), EGFR mutation (**C**), ALK-EML4 rearrangement (**D**), stage (**E**)**,** T (**F**)**,** N (**G**), and M (**H**) Stage. The univariate (**I**) and multivariate (**J**) Cox regression analysis of the associations between the risk score, clinical parameters and OS in NSCLC patients. NSCLC, non-small cell lung cancer; OS, overall survival

As shown in Fig. 8A–H, the results illustrated that risk score was related to gender, stage and T, N stage, among which, male patients and those with higher T and N stage had higher risk. Further subgroup analysis found that the T stage is correlated with risk score in both LUAD and LUSC, and stages and N stage were found to relate to the risk score in LUAD, and M stage showed a relationship with the risk score in LUSC (Additional file 1: Figure S5).

**Fig. 9:**
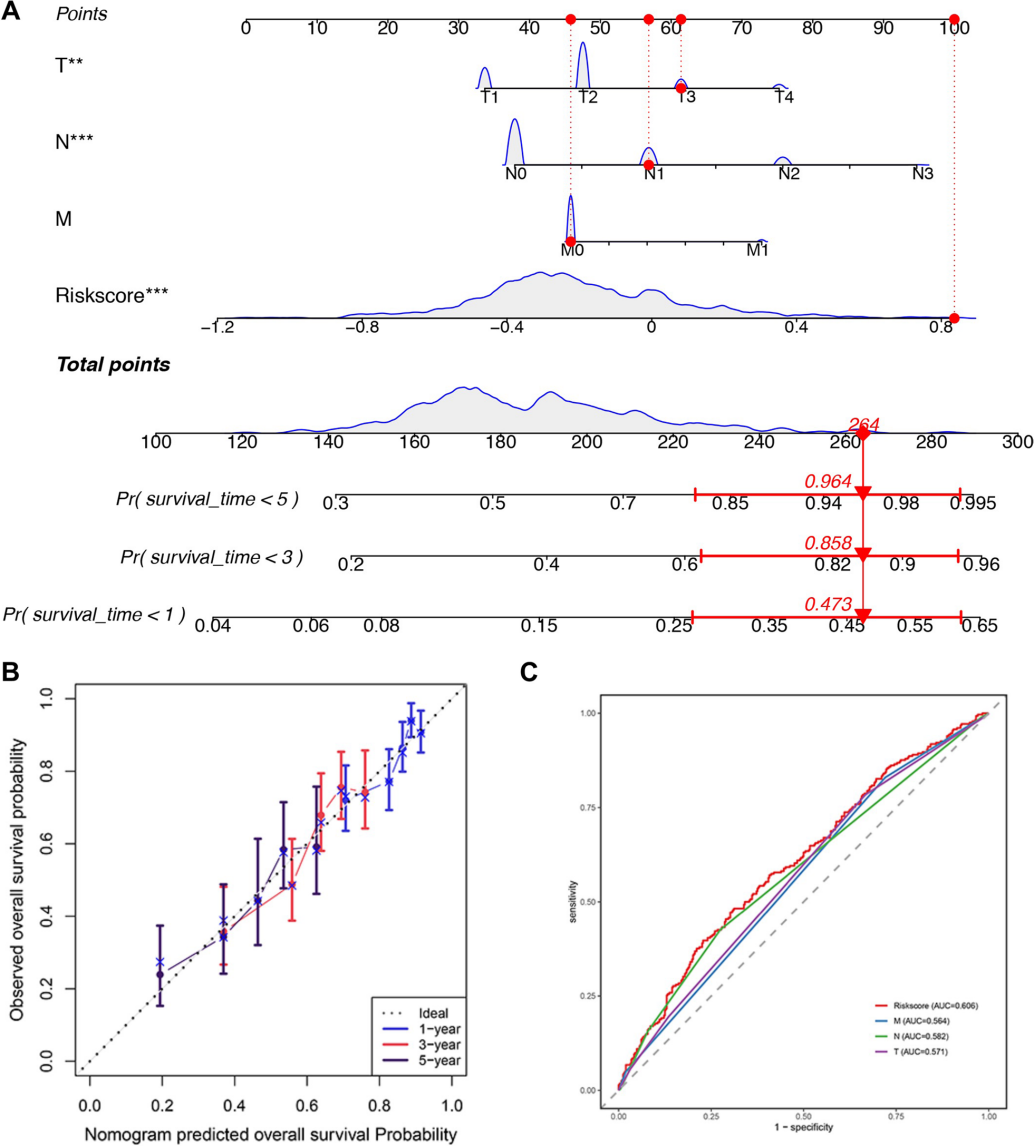
Nomogram construction and evaluation. (**A**) The nomogram based on the clinic-pathological factors and risk score. (**B**) The calibration curves of the nomogram in predicting 1-, 3‐ and 5‐years survival of NSCLC patients. (**C**) ROC curve of clinic-pathological factors and risk score for predicting prognosis of NSCLC. NSCLC, non-small cell lung cancer; OS, overall survival. FRGs, ferroptosis-related genes; FRGs-lncRNAs, FRGs-related lncRNAs; OS, overall survival

Univariate and multivariate Cox regression analysis elucidated that risk score, T, and N stages are independent risk factors for the OS of NSCLC patients (*P* < 0.05, Fig. 8I, J). Then, a nomogram merging independent risk factors (T, N and Risk score) and M stage with important clinical prognostic significance were constructed (Fig. 9A), and a satisfactory agreement between the predicted and observed values at the probabilities of 1-, 3- and 5-year survival was shown in the calibration curve (Fig. 9B). What’s more, the ROC curve revealed that the risk score was more accurate in predicting survival in patients with NSCLC than traditional clinical features (Fig. 9C).

### Correlation analysis between risk score, ICGs and chemotherapeutics sensitivity

**Fig. 10:**
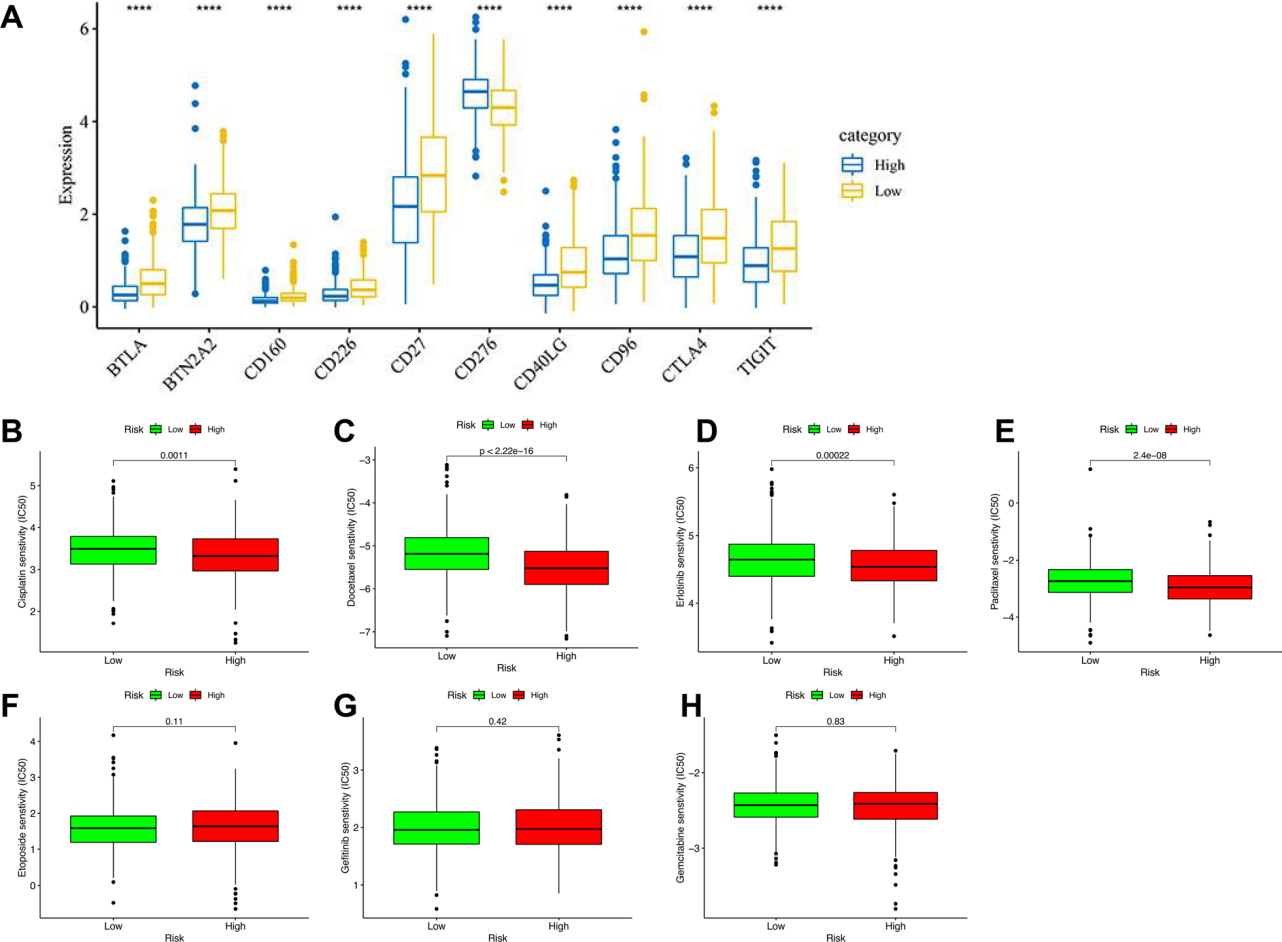
Correlation analysis between risk score, ICGs (**A**), and chemotherapeutics sensitivity (**B–H**) in NSCLC. ICGs, immune checkpoint genes; NSCLC, non-small cell lung cancer

The results showed a substantial difference in the expression of 45 ICGs between the two risk groups (Additional file 2: Table S6), and the first 10 ICGs (BTLA, BTN2A2, CD160, CD226, CD27, CD276, CD40LG, CD96, CTLA4, TIGIT) were presented in Fig. 10A. As shown in Fig. 10B–H, the correlation analysis between the risk score and the sensitivity of chemotherapeutics for NSCLC showed that patients with high-risk scores were highly sensitive to the chemotherapeutics cisplatin, docetaxel, erlotinib, and paclitaxel (all *P* < 0.05), while there was no significant difference in the sensitivity of the etoposide, gefitinib, and gemcitabine between the two groups (*P* > 0.05).

## Discussion

Lung cancer, the most fatal malignancy worldwide, has a variety of histological subtypes. As the most prevalent pathological pattern of lung cancer, NSCLC patients are usually diagnosed at an advanced stage with poor survival due to the lack of early specific clinical manifestations, diagnostic and prognostic biomarkers [31]. Over the past decade, the long-term survival rate of advanced NSCLC has been significantly prolonged due to the development of targeted therapies and immunotherapy. Unfortunately, poor prognoses remain in some patients after systemic and targeted therapy [32, 33]. Hence, there is an urgent need for safe and feasible markers that can accurately predict prognosis, so as to make the management of NSCLC patients more accurate, personalized and timely.

Ferroptosis is a newly type regulatory cell death with specific properties and recognizing functions that participated in numerous diseases including cancers [34], which was identified involved in killing malignant cells and inhibiting tumor progression in several cancers, such as NSCLC [35], pancreatic cancer [36], breast cancer [37] and hepatocellular carcinoma [38] and consequently considered as a novel therapeutic strategy for cancer treatment. Interestingly, the crucial role of lncRNAs in the regulation of ferroptosis has been increasingly recognized [39]. Whereas, the role of FRGs-lncRNAs in prognostic, immune response, and chemotherapeutic effect in NSCLC remains unclear.

Herein, we found 59 FRGs and 129 FRGs-lncRNAs differentially expressed between NSCLC and normal patients. Then, a prognostic model was established in the training set based on the 9 prognostic FRGs-lncRNAs and verified in the internal and external cohort. Additionally, the relationship between risk score and clinical characteristics of NSCLC were assessed and a nomogram was constructed. Finally, the correlation between ICGs, chemotherapeutic sensitivity as well as risk score was investigated to evaluate the potential role of FRGs-lncRNAs in the immune response and chemotherapeutic effect in NSCLC. These findings strongly suggest a potentially important role of FRGs-lncRNAs in NSCLC.

In our study, the predictive model was established based on the 9 FRGs-lncRNAs (DANCR, LINC00460, LINC00892, LINC00996, UCA1, and WWC2-AS2, AQP4-AS1, MED4-AS1, SNHG7,). Aquaporin 4 antisense RNA 1 (AQP4-AS1) transcribes a lncRNA with unknown function, which was found related to the risk of gastric [40] and breast cancer [41]. We found that AQP4-AS1 acts as a protector factor in NSCLC. Whereas, the biological function of AQP4-AS1 in ferroptosis and lung cancer has not been systematically analyzed, which needs to be further studied. LINC00460 was also demonstrated overexpressed in NSCLC and promotes epithelial-mesenchymal transition and cell migration [42], what’s more, as an FRGs-lncRNA, LINC00460 was identified as a predictor and potential therapeutic target for EGFR‑TKI resistance in NSCLC [43]. Although similar results with the previous studies were found in our study, whether ferroptosis involved in the drug resistance and development of NSCLC needs to be investigated in-depth. Despite several kinds of research that have illuminated the role of LINC00892 and LINC00996 in multiple cancers, their function in NSCLC has not been studied and deserves further investigation considering its significant prognostic value in NSCLC. Urothelial carcinoma-associated 1 (UCA1) was found to promote gefitinib-resistance in NSCLC [44], knockdown of UCA1 can impair cell proliferation and promoted the gefitinib-induced cell apoptosis, which was considered as a promising therapeutic target for the NSCLC patients with EGFR^+^ [44]. So far, no studies have been conducted on the biological function of WWC2-AS2 in NSCLC, which needs a systematic study in future. LncRNA differentiation antagonizing non-protein-coding RNA (DANCR) is known to suppress epidermal progenitor cell differentiation [45], which was identified to be overexpressed in multiple cancers, promoting malignant biological behavior cancer and chemo-resistance [46]. DANCR was also found upregulated and correlated with the poor prognosis in NSCLC, and which promotes the malignancy of NSCLC thorough DANCR/miR-138/Sox4 positive feedback loop [47]. MED4-AS1 is a novel lncRNA that is upregulated in NSCLC and is positively associated with poor differentiation and metastasis [48], The lncRNA small nucleolar RNA host gene 7 (SNHG7) was considered as an oncogenic lncRNA in NSCLC, hepatocellular carcinoma, breast cancer, and colorectal cancer [49, 50], which was found to modulate malignant character in LUAD through SNHG7/miRNA-181/cbx7 pathway, and mediates cisplatin-resistance in NSCLC through activating PI3K/AKT pathway [51]. Whereas, despite DANCR and SNHG7 being also found up-regulated in NSCLC in our study, both of them were found as protective factors, which may stem from the different genetic backgrounds, complications of patients with varying stages of NSCLC. In addition, different from the previous study [48], MED4-AS1 is a protective factor for the prognosis of NSCLC in the present study, which is due to the down-regulated expression of MED4-AS1 in NSCLC compared with normal tissue.

Recently, many studies have confirmed that ferroptosis not only plays an important role in the occurrence and development of tumors but also affects tumor immunotherapy [52, 53]. Induction of ferroptosis can enhance the effect of tumor immunotherapy. Hence, the expression of ICGs between the two risk groups was further investigated due to the crucial role of checkpoint inhibitor-based immunotherapies in NSCLC. We found different expression levels of BTLA, BTN2AA2, CD160, CD226, CD27, CD276, CD40LG, CD96, CTLA4, TIGIT between the two risk groups of patients with NSCLC. Of note, patients with high-risk scores were found highly sensitive to the chemotherapeutics cisplatin, docetaxel, erlotinib, and paclitaxel. These results indicated that these FRGs-lncRNAs may regulate the development and progression of NSCLC by modulating the immune response and play a crucial role in the drug resistance in NSCLC.

The advantage of this study is that the data of LUAD and LUSC were systematically combined for the analysis and an FRGs-lncRNAs prognostic model of NSCLC was constructed for the first time and verified both in an internal and external cohort. What’s more, the FRGs-lncRNAs were comprehensively identified both in the TCGA database and LncTarD database. Notably, the association between risk score and chemotherapeutics sensitivity was first analyzed in the present study. However, a prospective cohort and molecular biology experiment need to be conducted to further verify the accuracy of the prognostic model and the function of these FRGs-lncRNAs due to the lack of experimental verification in the present study.

## Conclusion

In conclusion, 10 FRGs-lncRNAs associated with the OS of NSCLC were identified and a novel FRGs-lncRNAs prognostic model was constructed and verified both in the internal and external cohort in the present study. Then, the relationship between ICGs, chemotherapeutics sensitivity and risk score were further evaluated. These findings have potential reference value for guiding the treatment and prognosis evaluation of NSCLC patients.

## Supplementary Information

**Additional file 1**. Supplementary figures.

**Additional file 2**. Supplementary tables.

## Data Availability

The data of this study were obtained from the publicly available database (https://xena.ucsc.edu/).

## Abbreviations

NSCLC: Non-small cell lung cancer
FRGs: Ferroptosis-related genes
LUAD: Lung adenocarcinoma
OS: Overall survival
LUSC: Lung squamous cell carcinoma
TCGA: The Cancer Genome Atlas
DE-FRGs: Differentially expressed FRGs
FRGs-lncRNAs: FRGs-related lncRNAs
GO: Gene ontology
KEGG: Kyoto Encyclopedia of Genes and Genomes
K-M: Kaplan–Meier plotter
ROC: Receiver operational characteristic
ICGs: Immune checkpoint genes
AQP4-AS1: Aquaporin 4 antisense RNA 1
DANCR: Differentiation antagonizing non-protein-coding RNA
SNHG7: Small nucleolar RNA host gene 7
UCA1: Urothelial carcinoma-associated 1
