# Supplementary material for: Identification of the ferroptosis-related long non-coding RNAs signature to improve the prognosis prediction and immunotherapy response in patients with NSCLC
Source: BMC Med Genomics. 2021 Dec 3;14:286. doi: 10.1186/s12920-021-01133-4 (PMC8642916; doi:10.1186/s12920-021-01133-4)
Supplement: Supplementary file 1 — Additional file 1. Supplementary figures. [file 12920_2021_1133_MOESM1_ESM.docx]

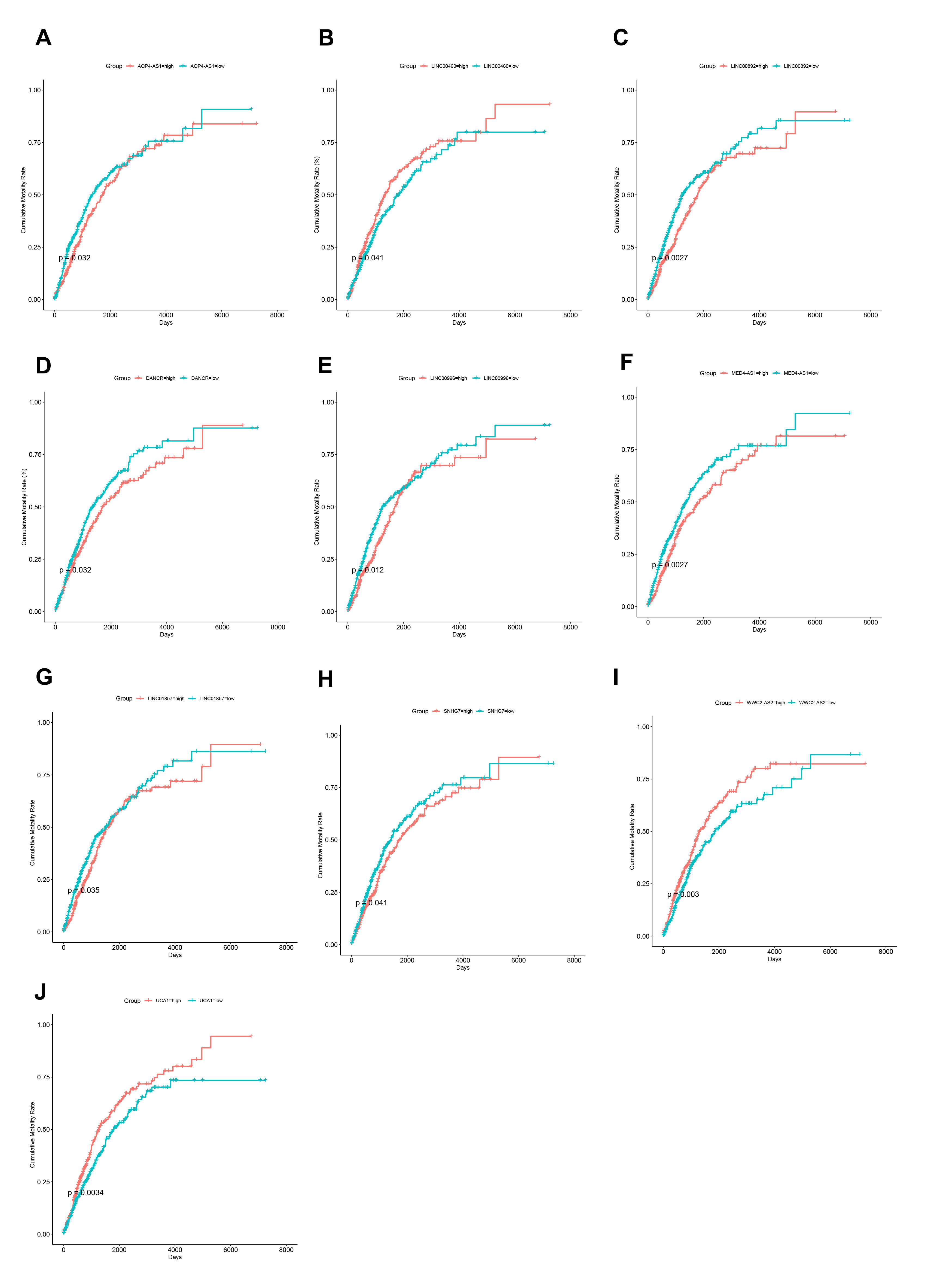


**Supplementary figure 1** eCDF plot of the prognostic FRGs-lncRNAs of NSCLC. eCDF, emperical cumulative density function. FRGs-lncRNAs: FRGs related lncRNAs, NSCLC, non-small cell lung cancer.


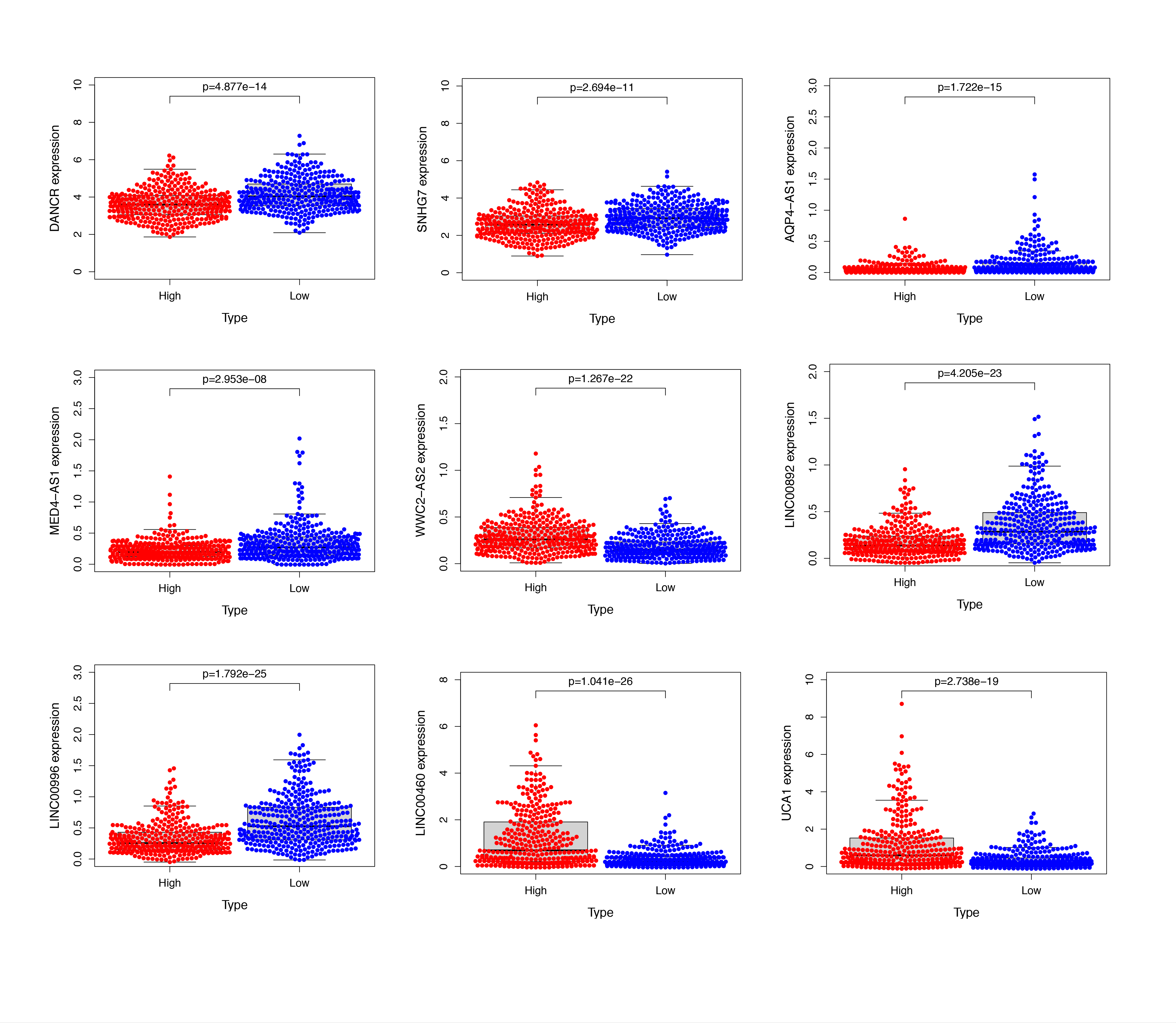


**Supplementary figure 2** The expression of the 9 FRGs-lncRNAs between the NSCLC patients with the high and low-risk groups in the training group. NSCLC, non-small cell lung cancer; lncRNAs: long non-coding RNAs; FRGs, ferroptosis-related genes; FRGs-lncRNAs: FRGs related lncRNAs.


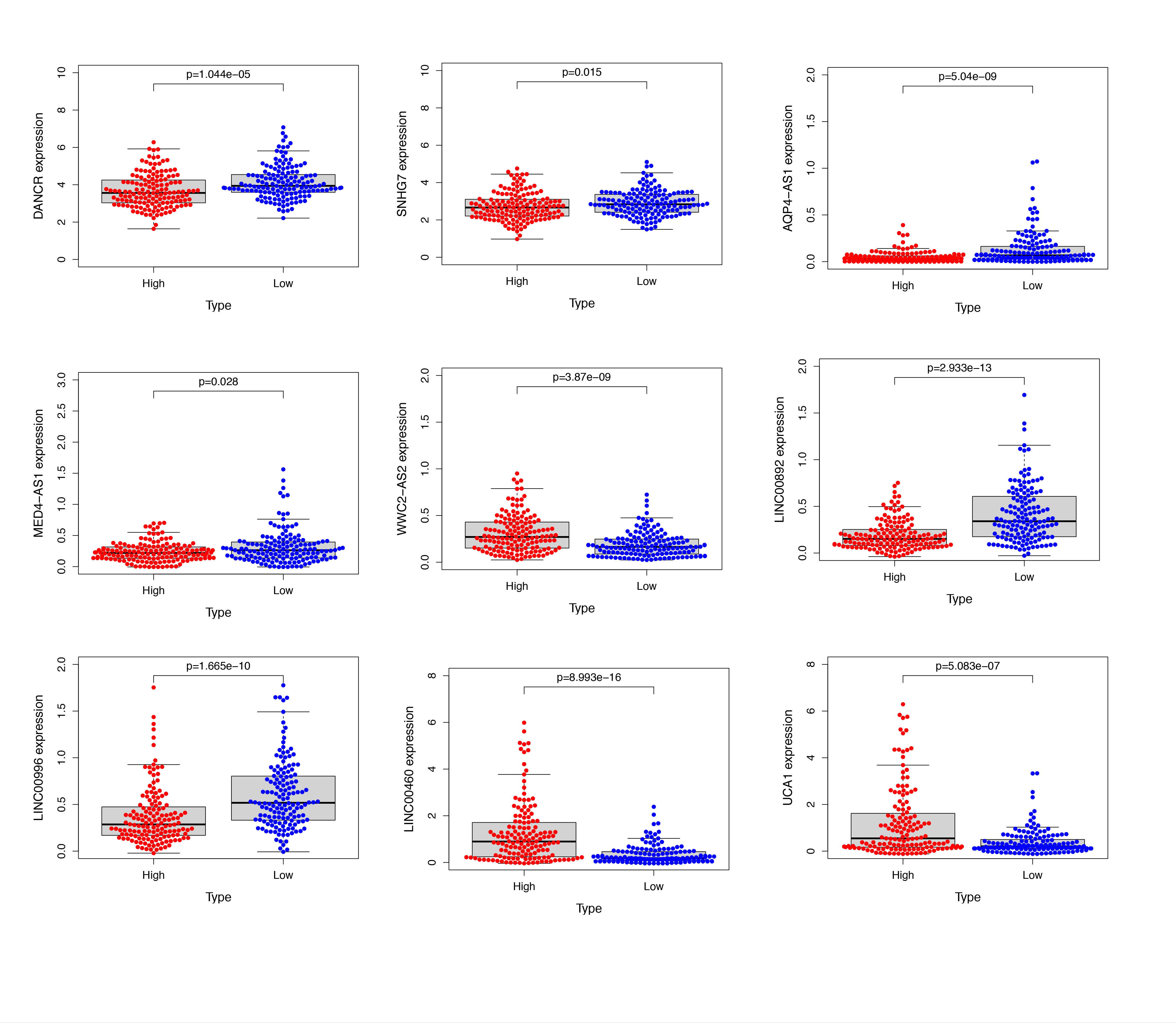


**Supplementary figure 3** The expression of the 9 FRGs-lncRNAs between the NSCLC patients with the high and low-risk groups in the testing group. NSCLC, non-small cell lung cancer; lncRNAs: long non-coding RNAs; FRGs, ferroptosis-related genes; FRGs-lncRNAs: FRGs related lncRNAs.


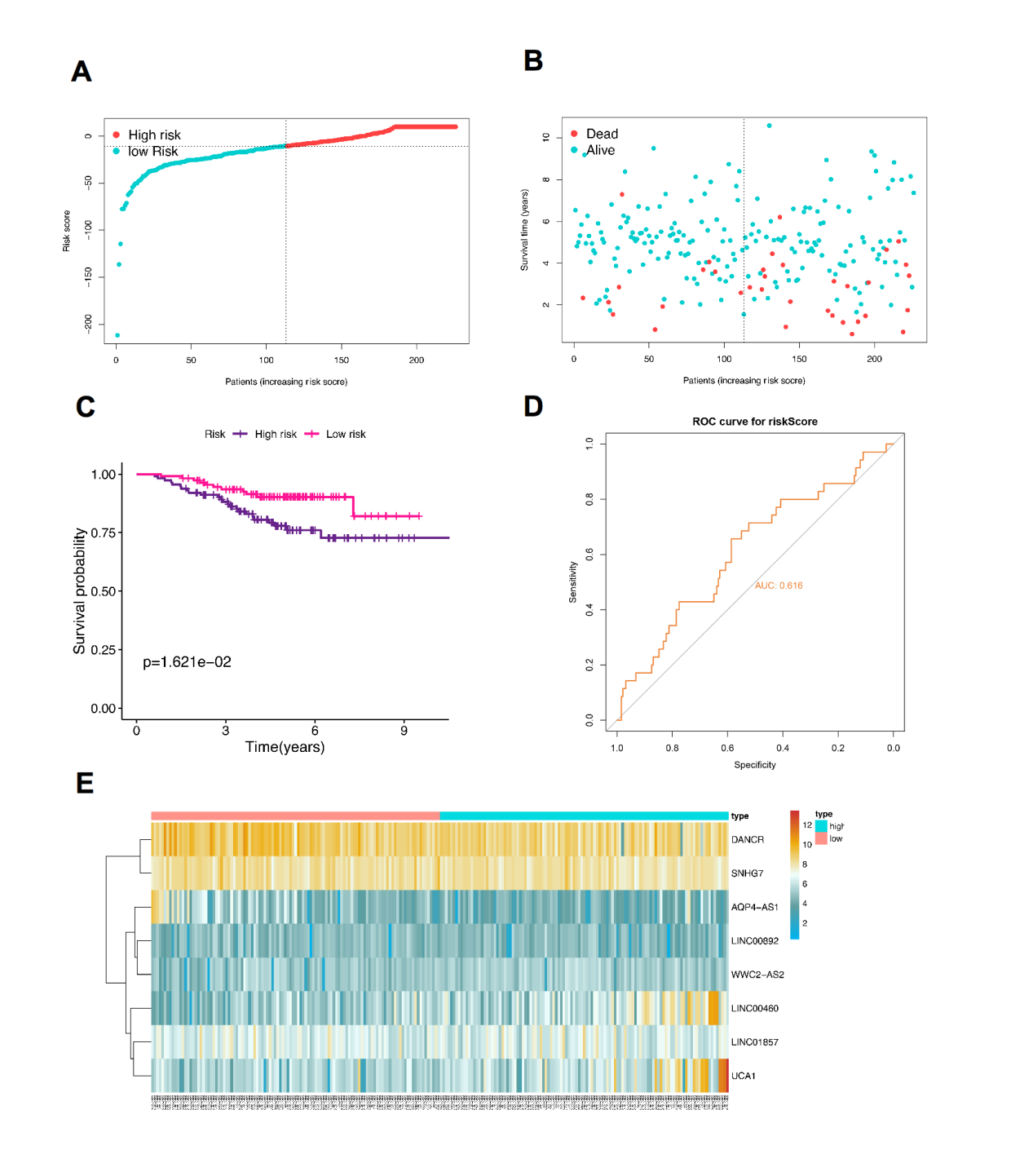


**Supplementary figure 4** External validation of the FRGs-lncRNAs signature. Distribution **(A)** and survival status plot **(B)** of NSCLC patients based on the median risk score. Kaplan-Meier survival **(C)** and ROC curve analysis **(D)** of the FRGs-lncRNAs signature in the external testing group. **(E)** Heatmap of the expression profiles of the FRGs-lncRNAs in low- and high-risk groups. NSCLC, non-small cell lung cancer; lncRNAs: long non-coding RNAs; FRGs, ferroptosis-related genes; FRGs-lncRNAs: FRGs related lncRNAs; ROC, receiver operating characteristic.


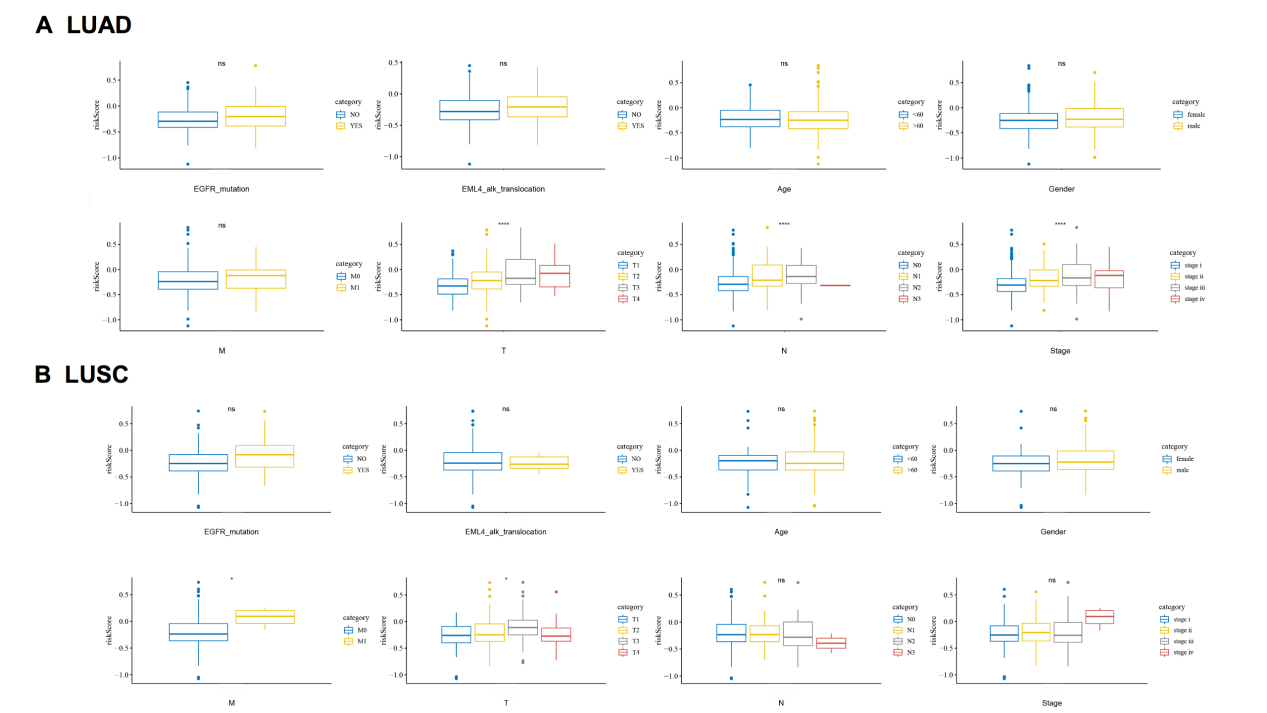


**Supplementary figure 5** Correlation analysis between clinicopathological features and risk score in LUAD **(A)** and LUSC **(B)**. LUAD: lung adenocarcinoma; LUSC: lung squamous cell carcinoma; OS: overall survival.
